# The impact of a closed-loop thalamocortical model on the spatiotemporal dynamics of cortical and thalamic traveling waves

**DOI:** 10.1038/s41598-021-93618-6

**Published:** 2021-07-13

**Authors:** Sayak Bhattacharya, Matthieu B. L. Cauchois, Pablo A. Iglesias, Zhe Sage Chen

**Affiliations:** 1 Department of Electrical and Computer Engineering, Whiting School of Engineering, Johns Hopkins University, Baltimore, MD 21218 USA; 2 Department of Mechanical Engineering, Whiting School of Engineering, Johns Hopkins University, Baltimore, MD 21218 USA; 3 Department of Psychiatry, Department of Neuroscience and Physiology, Neuroscience Institute, New York University Grossman School of Medicine, New York, NY 10016 USA

**Keywords:** Neuroscience, Computational neuroscience, Network models

## Abstract

Propagation of activity in spatially structured neuronal networks has been observed in awake, anesthetized, and sleeping brains. How these wave patterns emerge and organize across brain structures, and how network connectivity affects spatiotemporal neural activity remains unclear. Here, we develop a computational model of a two-dimensional thalamocortical network, which gives rise to emergent traveling waves similar to those observed experimentally. We illustrate how spontaneous and evoked oscillatory activity in space and time emerge using a closed-loop thalamocortical architecture, sustaining smooth waves in the cortex and staggered waves in the thalamus. We further show that intracortical and thalamocortical network connectivity, cortical excitation/inhibition balance, and thalamocortical or corticothalamic delay can independently or jointly change the spatiotemporal patterns (radial, planar and rotating waves) and characteristics (speed, direction, and frequency) of cortical and thalamic traveling waves. Computer simulations predict that increased thalamic inhibition induces slower cortical frequencies and that enhanced cortical excitation increases traveling wave speed and frequency. Overall, our results provide insight into the genesis and sustainability of thalamocortical spatiotemporal patterns, showing how simple synaptic alterations cause varied spontaneous and evoked wave patterns. Our model and simulations highlight the need for spatially spread neural recordings to uncover critical circuit mechanisms for brain functions.

## Introduction

Oscillatory neural activities in the brain that propagate across recording electrodes in space are called traveling waves. To date, macroscopic or mesoscopic traveling waves, interpreted as spatiotemporal neural dynamics, have been reported with various oscillatory frequencies (e.g., theta, alpha, beta, and gamma), spatial coverage (whole brain or local circuits), and modalities (e.g., slice physiology, multielectrode array, high-density EEG or ECoG, and voltage-sensitive dye optical imaging)^1–6^. As either neuronal spiking activity or field potentials from extracellular recordings, propagating waves have been found in a wide range of cortical, subcortical, and thalamic structures, and have been shown to be modulated in a spontaneous or task-dependent manner at in vivo or in vitro brain states^7–10^. However, investigating mesoscopic traveling wave patterns based on simultaneous multisite multielectrode-array recordings remains a challenge. Moreover, how these spatiotemporal patterns emerge because of intra- or inter-network connectivity of local neural circuits is still unclear. Biologically-inspired computational models, tightly linked to experimental data, provide a complementary approach to investigate these questions and offer new experimental predictions on the circuit mechanism of wave propagation^11–15^.

The thalamocortical network and thalamocortical oscillations play important roles in sensory processing, memory consolidation, and multisensory and sensorimotor integration^16,17^. Rhythmic or synchronous neural activity across various frequency bands has been observed in the thalamocortical system^16^. Propagating wave patterns have also been found in in vitro and in vivo recordings of thalamic and cortical areas at different brain states^4^. In contrast to continuous and smooth monosynaptic traveling waves observed in the cerebral cortex (CX), traveling waves often appear discontinuous (polysynaptic “lurching wave”) in the thalamus (TH), which involves the reciprocal interaction of excitatory thalamocortical (TC) and inhibitory GABAergic reticular nuclei (RE) cells^18–20^. In computational models computational models, traveling waves have been previously produced in an isolated thalamus or cortex structure, or in a one-dimensional (1D) thalamocortical system^18,21^. Nevertheless, the precise nature of how the complete thalamocortical structure, operated as a closed-loop system, determines traveling wave patterns is not completely understood. Additionally, whether the traveling patterns observed in a two-dimensional (2D) network may be preserved in the 1D projection remains an open question. To date, a wide range of models have been developed for traveling waves or spatiotemporal neural activity in neuronal networks^20,22–25^, neural fields^26–29^, networks of coupled oscillators^30,31^, and the thalamocortical network^32^. The modeling scale of network size varied between hundreds and tens of thousands of neurons^33^. The generation of diverse wave patterns—similar to those seen in experiments—mostly relied on neural field continuum approximations^34^.

The different layers of the thalamocortical system serve as *excitable* systems capable of sustaining diverse wave patterns. Previous studies have reported that controlling different parameters of an excitable system, including *time-scale separation* (the delay between activator and inhibitor rise-times), *space-scale separation* (faster spreading inhibitor than the activator), and *threshold* (excitation-inhibition balance) can have drastic effects on spatiotemporal patterns^35^. A multi-layered neural circuit, thus, provides an excellent canvas for such pattern generation. For example, the space-scale separation may simply amount to inhibition from one cortical layer that spreads faster than another through divergent connections^32,36^. Similarly, time-scale separation can be equivalent to the conductions delay between long-range connections. Each neuron has an activation threshold, which can be easily altered by changing the balance between excitatory and inhibitory synaptic strengths. Thus, by indirectly manipulating these system parameters, it may be possible to generate and control a wide range of complex spatiotemporal wave patterns, such as the spiral waves during sleep^5^, the repeating planar waves during visual responses/perception tasks^10,37^, or the bimodal waves propagating in opposite directions during motor tasks^3,6^.

Here, we develop a computationally efficient network model of the 2D topographic thalamocortical structure that produces dynamic spatiotemporal patterns from the closed-loop interaction of a total of 10,800 cortical and thalamic cells. While our proposed network is a reduced version of realistic thalamocortical circuits simplifying several biological details, it focuses on other important factors, such as the intracortical connectivity, excitation/inhibition (E/I) imbalance, thalamocortical (or corticothalamic) delay, and their impact on the spatiotemporal traveling waves. Our model demonstrates that rich spatiotemporal patterns can emerge independently or jointly from the interactions of these contributing factors. Moreover, our model shows that spatiotemporal patterns and characteristics that are commonly observed in different brain states and behavioral tasks can be interchangeably controlled through alterations in the aforementioned model parameters, even without the use of complex neural-field approximations^34^. Furthermore, by modulating specific intracortical or thalamocortical connection weights and thalamocortical or corticothalamic delay parameters, the model produces a diverse range of wave patterns that may be indiscernible without two-dimensional (2D) recording techniques.

## Results

### A closed-loop thalamocortical model architecture sustains propagating waves and oscillations

**Figure 1:**
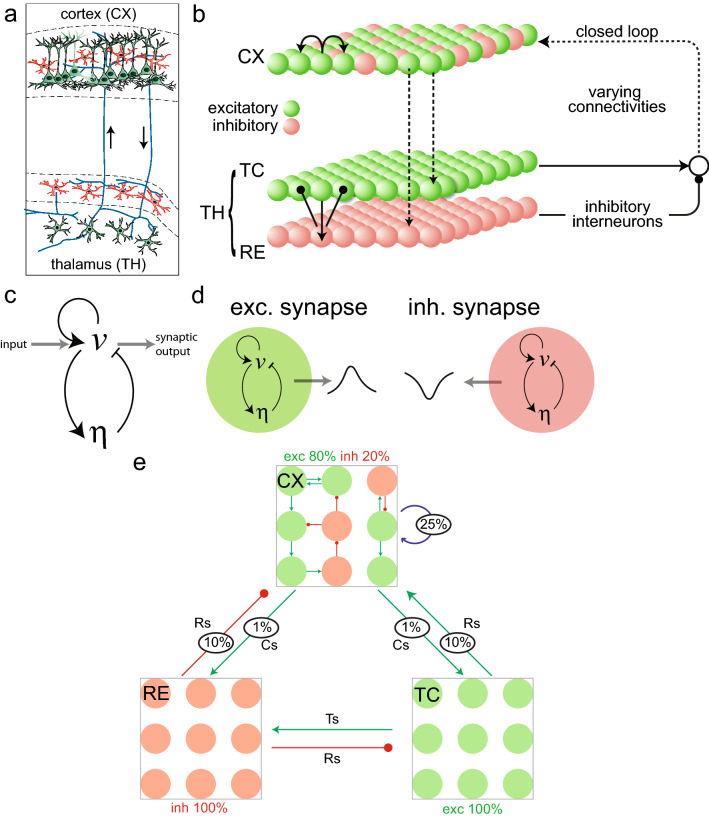
Neuron model and thalamocortical network structure. (**a**) Diagram of the thalamocortical circuit (adapted by permission from: Destexhe A., Contreras D. The fine structure of slow-wave sleep oscillations: from single neurons to large networks. In: Hutt A. (eds) Sleep and Anesthesia. Springer Series in Computational Neuroscience, vol 15. Springer, New York, NY, copyright (2011)^39^). (**b**) Two-dimensional (2D) schematic representation of the computational model with a three-layer architecture: CX (cortex, containing both excitatory and inhibitory cells, size: 60 $\,\times\,$ 60), RE (inhibitory thalamic reticular nuclei cells, size:60 $\,\times\,$ 60) and TC (excitatory thalamocortical relay cells, size: 60 $\,\times\,$ 60). RE-TC are reciprocally connected. The network is connected in a closed loop. (**c**) The excitable neuron module, showing the self-enhancing voltage term (*v*) with negative feedback from the gating variable ($\eta$), and the input-output scheme (**d**) Schematic showing how each module can be excitatory (green) or inhibitory (red) depending on the type of synaptic output. (**e**) Graphical illustration of thalamocortical network connections, with green indicating excitatory (exc), red indicating inhibitory (inh), and blue indicating mixed excitatory and inhibitory connections, respectively. $T_s$, $R_s$, and $C_s$ represent the synapses for the TC, RE, and CX layers, respectively. The number inside the circle represents the connectivity percentage used in the normal operating mode.

We developed a model of the thalamocortical network consisting of one layer representing cells from the cortex (CX) and two layers of the thalamus: the excitatory thalamocortical relay cells (TC) and the inhibitory thalamic reticular nuclei cells^18^ (RE) (“Methods”; Fig. 1a). Each layer consists of a 60 $\,\times\,$ 60 two-dimensional (2D) array of neurons (Fig. 1b). While it is well known that the cortex is a multi-layered structure with complex and potentially long-range interconnections^32^, for the sake of simplicity we collapsed the multiple laminar structure into a single-layer structure. In each layer, every neuron is modeled using a set of differential equations representing a two-state excitable system (Fig. 1c). One state, the *voltage term* (*v*), has self-enhancing positive feedback and is analogous to the neuron’s membrane potential. The other is the *gating variable* ($\eta$) which provides negative feedback and brings the voltage back to resting equilibrium. This two-state system has an activation threshold (owing to a bifurcation point close to the equilibrium, see **“Methods”) which when crossed generates a spike in activity (Supplementary Fig. S1a). This threshold depends on the system parameters and can also be modulated with external inputs (“Methods”). The two-state dynamical system approximates a spiking neuron by assuming instantaneous activation of the sodium current (contributing to the fast voltage term) and a slower potassium inactivation (gating variable)^38^. The differential equations and the parameter values (presented in Tables 1 and 2) of the thalamic and cortical neurons were chosen to make each neuron excitable but differ slightly based on the frequency of firings that each type of neuron is known to generate^38^ (“Methods”).

**Table 1:** A tabular summary of computer simulation setup and parameters for the three-layer thalamocortical system.

Parameter	Description	Setup
*N*	Number of neurons	$60\times 60$
$i_p$	Inhibitory synapse parameter	5
$k_n$	Intra-cortical connectivity weight	16.2
$(s_1, t_1)$	Synapse equation parameters	(10, 0.01)
$(a_1, a_2, a_3, a_4, a_5)$	Voltage equation parameters	(0.167, 16.67, 167, 1.2, 1.47)
$(c_1, c_2)$	Gating equation (Type-3) parameters	(0.05, 1.5)
$(d_1, d_2, d_3, d_4)$	Gating equation (Type-1) parameters	(0.09, 0.6, 0.3, 0.18)
$w_{\mathrm {RE}\text{- }\mathrm {TC}}$	Weight from RE to TC	3
$w_{\mathrm {TC}\text{- }\mathrm {RE}}$	Weight from TC to RE	12
$w_{\mathrm {RE}\text{- }\mathrm {CX}}$	Weight from RE to CX	0.02
$w_{\mathrm {TC}\text{- }\mathrm {CX}}$	Weight from TC to CX	25
$w_{\mathrm {CX}\text{- }\mathrm {TC}}$	Weight from CX to TC	0.75
$w_{\mathrm {CX}\text{- }\mathrm {RE}}$	Weight from CX to RE	7.5
$w_E$	Weights for excitatory neurons in CX	3
$w_I$	Weights for inhibitory neurons in CX	6
$M_{\mathrm {sparse}}$	Bnary sparsity matrix for CX-RE/CX-TC connections	1% connected (random)
$W_{\mathrm {sparse}}$	Binary sparsity matrix for RE-CX/TC-CX connections	10% connected (random);
$X_{\mathrm {sparse}}$	Binary sparsity matrix for intra-cortical connections	symmetric
$C_{\mathrm {TH}}$	Binary sparsity matrix for 4-type connectivity	
$\mu$, $\sigma$	Gaussian noise parameters	0, 0.1

**Table 2:** Changes of parameters for a reduced CX-TH thalamocortical system. All other parameters remain the same as in Table 1. All parameters for the TC layer are ignored.

Parameter	Description	Setup
$k_n$	Intra-cortical connectivity weight	9.72
$(c_1, c_2)$	Gating equation (Type-3) parameters	(0.2, 0.6)
$(d_1, d_2, d_3, d_4)$	Gating equation (Type-1) parameters	(0.3, 2, 0.3, 0.6)
$w_{\mathrm {TH}\text{- }\mathrm {CX}}$	Weight from TH to CX	7
$w_{\mathrm {CX}\text{- }\mathrm {TH}}$	Weight from CX to TH	1
$w_E$	Weights for excitatory neurons in CX	6
$w_I$	Weights for inhibitory neurons in CX	6
$w_{\mathrm {TH}}$	Lateral excitatory weight in TH layer	15
$W_{\mathrm {sparse-TH}}$	Binary sparsity matrix for RE-CX/TC-CX connections	10% connected (random);

Additionally, each neuron has a synaptic output equation that describes its effect on its neighbors, which can be either excitatory or inhibitory, depending on its effect on the post-synaptic neurons (green or red, respectively, in Fig. 1d). We assume that every neuron in TC is excitatory and that every neuron of RE is inhibitory. In contrast, neurons in the cortex layer are randomly chosen from both classes to keep the composition at 80% excitatory and 20% inhibitory^33^ (Fig. 1e). Every neuron of the CX could be connected to four different neurons (a fully connected layer). The overall CX connectivity was varied for different simulations. In simulations when the connectivity was less than 100%, random connections were removed until the desired level was reached (“Methods”, Fig. 1e).

In addition to connections between nearest-neighbor neurons in each layer, neurons across different layers are also connected. The cortex and the thalamus are connected through bottom-up (feedback) and top-down (feedforward) connections^39^ (Fig. 1b). Within the thalamic layers, the RE and TC neurons are connected reciprocally such that activity progresses through the layers via mutual recruitment of neurons^18^. Specifically, neurons from the RE layer inhibit TC neurons which cause a rebound spike^18^ after a delay. The TC neurons then excite RE neurons via axon collaterals, generating further post-inhibitory rebound spikes, thus repeating the process. We assume that 10% of excitatory (TC) and inhibitory (RE) neurons affect the cortex directly, and 1% of the cortical neuron (excitatory) feedback to both thalamic layers—thus closing the thalamocortical loop (Fig. 1e).

**Figure 2:**
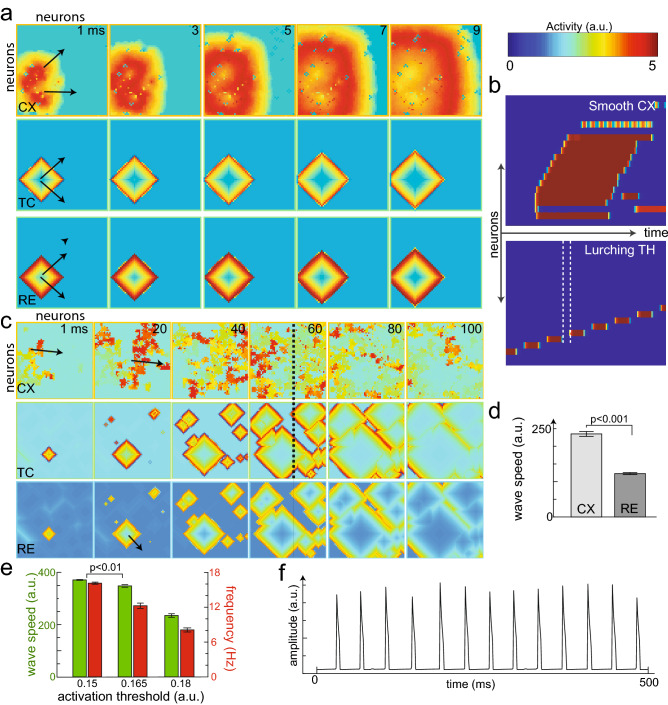
Thalamocortical model and simulated traveling waves. (**a**) Traveling waves produced by the model operated in an open loop (i.e., without CX$\rightarrow$RE connection, 99% excitatory cortical neurons). Each square shows the 60$\,\times\,$60 array layout of neurons, and colors indicate the level of activity observed. Dynamic traveling wave patterns are shown (assuming a dense intracortical connectivity); arrows indicate wave directions. Color bar show the scale of neuronal activity (a.u.). (**b**) One-dimensional (1D) projection of the traveling waves to indicate the different wave dynamics between the thalamus and the cortex. The gap between the white dashed lines in the thalamus shows the lurching pattern as the wave is staggered in time; in contrast, the cortical wave is smooth. (**c**) Same layout as a. Computational model operated in a closed loop (with 1% CX$\rightarrow$TH connections and 80% excitatory neurons) results in oscillations with random wave directions. The black dashed line denotes the region from which the 1D project in b was taken. (**d**) The average cortical wave speed was significantly faster than the thalamic wave speed ($p<0.0001$ from five simulations, Student’s* t* test). Error bar represents standard error of mean (SEM). (**e**) Bar graphs showing how traveling wave speed and frequency is altered with system threshold (parameter d__4_ cortical gating equation in “Methods”). *p* values were computed from 5 simulations (Student’s* t* test). (**f**) Time oscillations produced by the cortex of around 15 Hz frequency.

In vitro experiments have shown that cortical waves are smooth, while thalamic waves are staggered in time^4^. To validate the model, we first examined traveling wave patterns in a simplified open-loop setting (i.e., without $\mathrm {CX}\rightarrow \mathrm {RE}$ and $\mathrm {CX}\rightarrow \mathrm {TC}$ connections, assuming nearly fully connected or $\,\sim \,$99% intracortical connections, 99% excitatory neurons). Our 2D thalamocortical model produced traveling waves in both the cortex and thalamus (Fig. 2a). In a 2D graphical illustration, the dynamic evolution of traveling waves was visible in time (from left to right panels, with arrows indicating the wave direction). Furthermore, the mutual recruitment of excitatory spikes and delayed rebound spikes created the lurching wave phenotype in the thalamus^19^, resulting in periodic gaps of temporal activity (Fig. 2b), in direct contrast to the smooth cortical wave. The traveling wave initiated at a specific location in the 2D topological space, then the wave pattern spread in space (see 1D projection in Fig. 2a). Next, we examined traveling waves in a closed-loop setting (i.e., with $\mathrm {CX}\rightarrow \mathrm {RE}$ and $\mathrm {CX}\rightarrow \mathrm {TC}$ connections restored), based on a similar connectivity setup. Our model produced sustained oscillations in both the thalamus and cortex (Supplementary Video S1). Notably, this was a deterministic simulation (changing the initial condition of a neuron in the cortical sheet) without any stochastic input to cortical neurons. Interestingly, periodic oscillations were sustained because of the closed-loop feedback and feedforward connections, although the system operating condition was away from the bifurcation point (Supplementary Fig. 1e). That is, the emergent oscillations were not the result of a limit cycle due to the instability of the equilibrium^40^, but rather the consequence of the closed-loop structure, without which the thalamic oscillations failed to persist (Supplementary Fig. S1f). Supplementary Video S2 provides a “zoomed-in” view of the spontaneous wave propagation simulated in the cortex, showing a 10$\,\times\,$10 array instead of a 60$\,\times\,$60 array to match experimentally observed structures. Next, we modified the model to contain 80% excitatory and 20% inhibitory cortical neurons to match ratios seen experimentally^33,41^; we also assumed 99% intracortical connectivity, with stochastic inputs. In this case, our model produced oscillations with spontaneous random wave directions (Fig. 2c and Supplementary Video S3. Supplementary Video S4 shows a zoomed-in 10$\,\times\,$10 array). The 1D projections illustrate the effect of inhibitory neurons that disrupted the cortical waves by creating inaccessible regions (Supplementary Fig. S1g). On average, the cortical wave speed was faster than the thalamic wave speed (Fig. 2d). This, of course, was due to the monosynaptic nature of the cortical waves as opposed to the polysynaptic thalamic waves that were staggered in time (owing to the delayed post-inhibitory rebound spike), allowing two different wave patterns to sustain in a closed-loop manner. Lowering the activation threshold of the cortical neurons (“Methods”, Supplementary Fig. S1c, bottom) increased the wave speed, as expected from singular perturbation studies of excitable systems^42^. Additionally, it increased the driving input to the closed-loop system changing the oscillation frequency (Fig. 2e). Therefore, the system threshold could be used to couple wave speed and frequency resulting in a mutually positive correlation. The operational threshold was chosen such that the oscillations occurred in the 10-20 Hz range, typical of alpha (lower beta) rhythms in the cortex^2–4^ (Fig. 2f). Furthermore, we set the feedback connectivity between the cortex and thalamus around 1%. A higher degree of $\mathrm {CX}\rightarrow \mathrm {RE}$ or $\mathrm {CX}\rightarrow \mathrm {TC}$ connectivity caused the cortical wave to dominate the thalamic wave, causing the thalamic lurching (staggered activity in time) to vanish quickly (Supplementary Video S5 and Supplementary Fig. S2a). Together, these results suggest that in contrast to open-loop—the closed-loop thalamocortical model produces rich oscillatory activity with random traveling wave patterns and sustains distinct traveling wave speed/patterns between the cortex and thalamus.

### Low intracortical connectivity necessitates clustered cortical neurons to yield traveling waves

**Figure 3:**
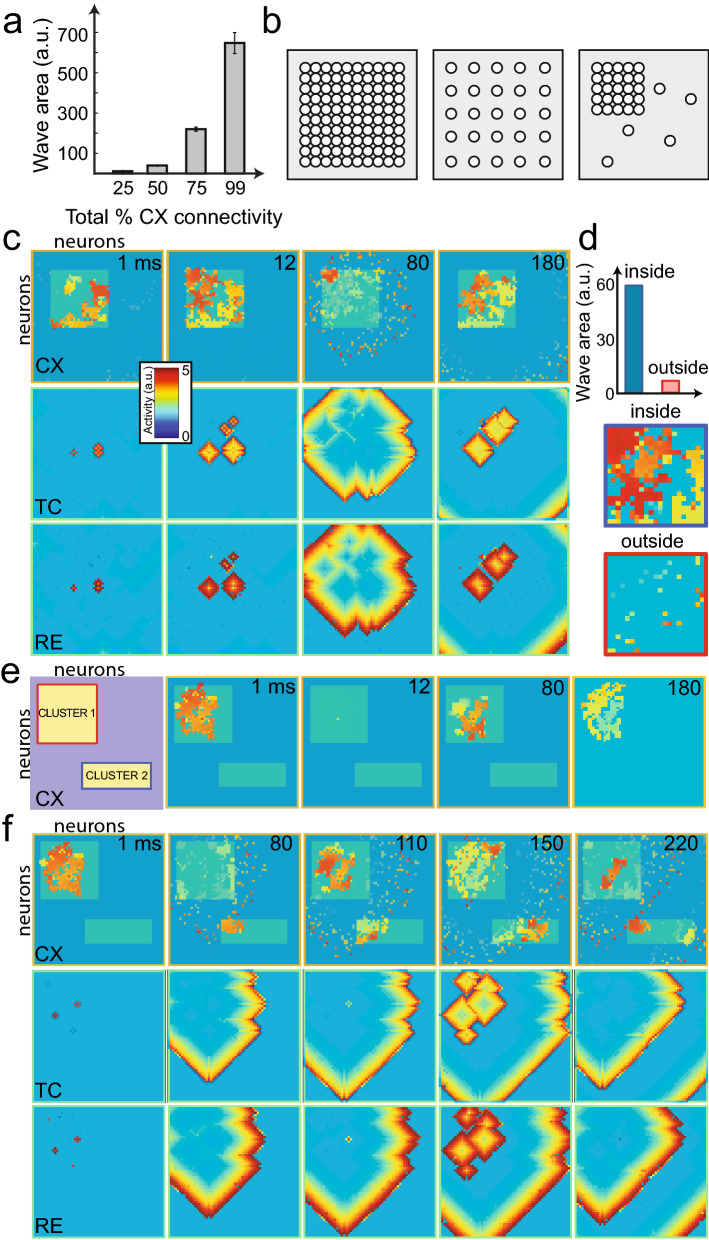
Network connectivity controls patterns of spontaneous traveling waves. (**a**) Traveling wave area was reduced with decreasing overall intracortical connectivity. Error bar represents SEM from 5 simulations. (**b**) Schematic showing different CX arrangement (neuron array): fully connected (left), uniformly connected with lower connectivity (middle), and clustered and with the same overall connectivity (right). We assumed that the RE and TC layers have uniform arrangement. (**c**) Traveling waves produced by the model operated in a closed loop (with overall 25% intracortical connectivity and a 90% intra-connected cluster). The unshaded portion in the CX illustrates the clustered region. Each panel is the 60$\,\times\,$60 neuron array layout, and colors indicate activity levels. (**d**) Comparison of traveling wave area between the inside and outside the clustered region. The wave activity was prominent only within the cluster (zoomed in a snapshot via a blue box), whereas only puncta-type activity was seen outside the cluster (zoomed in a snapshot via a red box). (**e**) Disconnected thalamocortical network with only CX setting: two clusters, both 99% connected, with overall 31% intracortical connectivity. The cluster positions are shown on the leftmost panel. The activity was triggered stochastically within the red cluster. Because of the weak connectivity between the two clusters, the activity in the red cluster did not reach the blue cluster. (**f**) Same as panel e, except with the thalamus connected in a closed loop. The thalamic wave enabled communications between the two clusters.

To sustain wave propagation, or equivalently, to maintain sufficient wave propagation area in time, we found that a high percentage of intracortical connectivity was required. In the simulations, we varied overall intracortical connectivity between 25–36%^41^, with 4:1 ratio of excitatory to inhibitory cortical neurons. The area of the traveling wave was calculated as the number of neurons engaged in the wavefront. In the cortex alone, with 25% intracortical connectivity, the traveling wave area was low. That is, we found that it was difficult to sustain spontaneous cortical wave propagation with 25% connections. The cortical wave area increased proportionally with the intracortical connectivity (Fig. 3a), reaching a noticeable wave structure when the intracortical connections reached $\,\sim \,$50%; this was consistent with a previous report using another model setup^33^. When the intracortical connectivity was below 25%, the cortical wave structure lost continuity and reduced to isolated dot patterns. Next, we added the thalamus in the closed loop, and observed a punctate wave band in the cortex, completely in synchronization with the thalamus (Supplementary Fig. S2b), thereby not enabling spontaneous cortical wave propagation. This motivated us to incorporate clustered intracortical connections to the model, with a goal of sustaining traveling waves.

**Figure 4:**
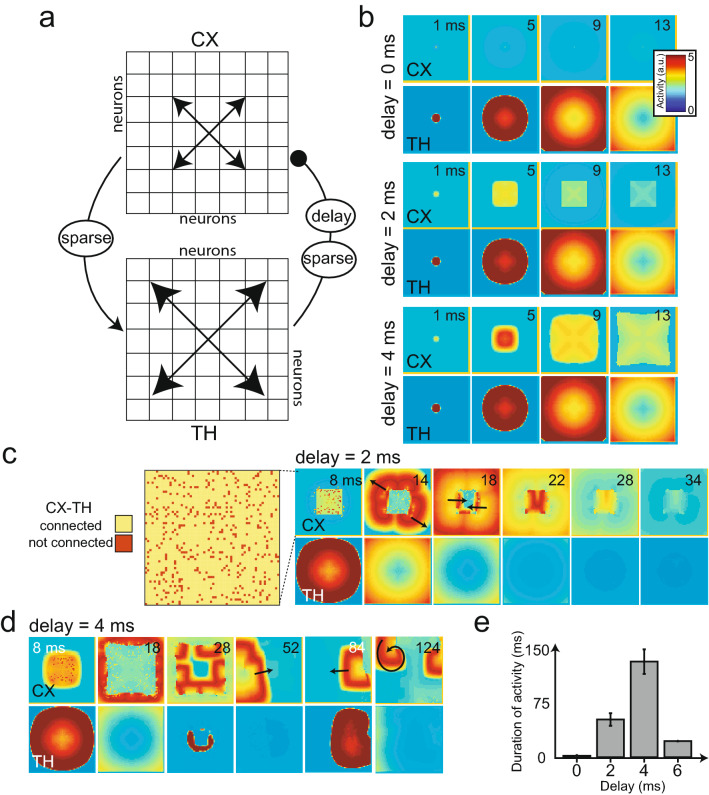
Transmission delay between the cortex and thalamus changes the stimulus-evoked traveling wave patterns. (**a**) A reduced thalamocortical model showing interactions between CX and inhibitory TH cells with lateral connections. The grid denotes the 2D neuron array layout. A nonzero delay parameter was introduced between TH and CX connection to account for axonal conduction delays. We assumed that CX was fully connected with purely excitatory neurons. (**b**) Impact of different thalamocortical delay parameters on the CX wave dynamics (assuming fully connected TH-CX). With an increased delay, the CX wave could propagate further and longer. In contrast, lateral excitation allowed the TH wave to propagate unrestricted regardless of the delay. Colors denote activity levels. (**c**) A 90% connected TH-CX condition, where the red dots denote the cortical neurons that receive no TH inhibition. For a specific thalamocortical delay of 2 ms, the uninhibited points produced a new CX wave that propagated in various directions (indicated by black arrows), and TH wave activity ultimately disappeared. (**d**) With an increased delay of 4 ms, dynamic wave activity emerged. In this illustration, radial ($t=8$ ms), planar ($t=52$ ms), and rotating ($t=124$ ms) waves were produced. (**e**) Comparison of the wave activity duration with respect to different delay parameters. Their non-monotonic relationship suggests an optimal delay regime in the thalamocortical network. Error bar represents SEM.

In cortical circuits, excitatory connections are not uniformly distributed, and often form clustered groups of highly connected neurons^41^. A low overall percentage of intracortical connectivity may arise from high connectivity within clustered groups, with much lower connections outside. To investigate the impact of connectivity topography, we modified the 2D arrangement of neurons from uniform connectivity (Fig. 3b, left and middle panels) to a more clustered structure (Fig. 3b, right panel, with the same overall connectivity as the middle panel) (“Methods”). Within the clustered group, the intracortical connectivity was $\,\sim \,$90%, while maintaining the overall 25% connectivity. Simulations with this setting were able to sustain propagating waves within the cluster, as opposed to punctate patterns outside the cluster (Fig. 3c,d).

Furthermore, we compared the impact of open vs. closed-loop on cortical traveling waves in a clustered setup. In the open-loop setting, we assumed that there was 99% intracortical connectivity within two clustered cortical neuronal groups (with overall 31% connectivity). The cortical wave was initially triggered within cluster 1 but failed to propagate to cluster 2 (Fig. 3e). In contrast, in the closed-loop setting, under the same 31% connectivity condition, propagating waves were present in both the thalamus and two cortical clusters (Fig. 3f and Supplementary Video S6). This result suggests a potential role of the thalamus and thalamocortical connections in communicating cortical traveling waves across multiple isolated cortical areas.

### Thalamic connections and thalamocortical delay reshape spatiotemporal cortical dynamics

Inhibition plays a key role in shaping spatiotemporal patterns^35^. To explore the diversity of cortical waves that we could generate, we focused on the impact of thalamic inhibition on the cortex^43^. To simplify the model, we collapsed the thalamus into one layer (TH) that contained strong intra-thalamic excitation, which in turn inhibited the cortex (CX) (Table 2, Fig. 4a). This effect is thought to occur through the intermediate inhibitory interneurons^43^, which have not been explicitly considered in our model. To remove potentially confounding effects of inhibitory cortical neurons, we made a simplifying assumption that CX was fully connected with excitatory neurons only, analogous to the zoom-in view of a clustered group. We assumed full TH-CX connectivity and introduced a delay parameter between TH and CX (i.e., no instant feedback), as the time delay during synaptic transmission within a closed-loop system is known to play an important role in its intrinsic dynamics^25,44^.

The axonal conduction delay is known to be in the order of milliseconds^43^. Therefore, we systematically varied the thalamocortical delay parameter (0, 2, 4 ms) and observed the change of spatiotemporal wave patterns in TH and CX (Fig. 4b). When there was no delay and TH-CX were fully connected, the cortical wave was instantly disrupted by thalamic inhibition. In contrast, introducing a 2 ms delay allowed the cortical wave to propagate to a certain distance before a complete disruption by thalamic inhibition. The distance that the cortical wave could travel before being subdued defined the “cortical firing field”. An increase of the delay from 2 ms to 4 ms further expanded this firing field, i.e. enabled the cortical wave to propagate further.

Next, in the reduced model, we decreased the TH-CX connectivity percentage from 100% to 90%, where the unconnected neurons were chosen randomly (Fig. 4c, leftmost panel). In the case of an intermediate delay, a rich repertoire of cortical traveling wave patterns, including radial, planar, and rotating waves was obtained (Fig. 4c,d and Supplementary Video S7 and Supplementary Video S8). We observed that the traveling wave direction or pattern could spontaneously change in time (c.f. $t=14$ vs. 18 ms in Fig. 4c; $t=52$ vs. 84 vs. 124 ms in Fig. 4d). Additionally, the duration of the cortical wave pattern depended on the delay parameter (Fig. 4e). A small delay led to a quick disruption of cortical waves because of thalamic inhibition, whereas a large thalamocortical delay caused the cortical wave to escape the field of view before thalamic inhibition became effective. Together, these results suggest that an optimal delay regime may exist to maintain the cortical traveling wave structure for a thalamocortical network with specific connectivity.

### Thalamocortical connectivity controls cortical and thalamic wave patterns and characteristics

**Figure 5:**
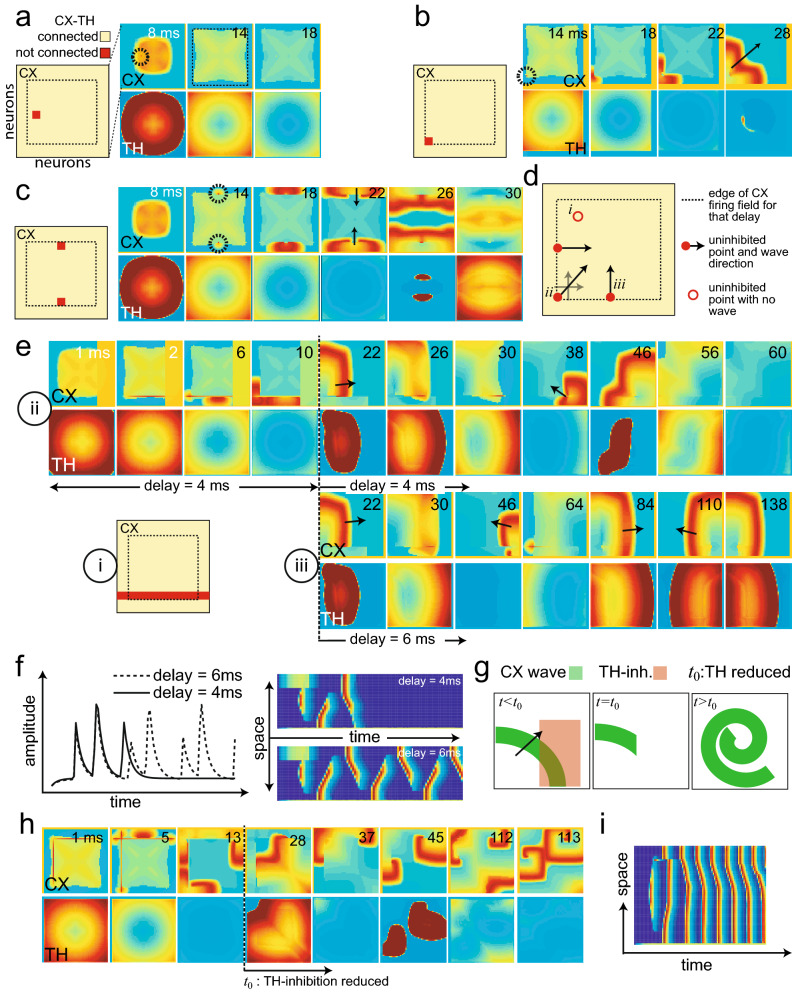
TH-CX connectivity and thalamocortical delays determine cortical and thalamic wave patterns. (**a**) Left: A zoomed-in CX circuit showing neurons (red points) that are disconnected to TH. Right: Traveling wave dynamics in the CX and TH with assumed lateral intra-TH connections. The black circle indicates the uninhibited point. When this uninhibited point fired, it could not produce a wave because of the surrounding CX refractory zone that received TH inhibition. (**b**) Similar to panel a, except the unconnected point was located at the edge of the CX firing zone (smaller dashed box). The black arrow shows the wave direction. (**c**) Changing unconnected point locations altered wave directions. (d) Schematic summarizing how the location of the uninhibited nodes produces traveling waves with various directions. (**e**) i: The unconnected points were in a straight line so that the resulting wave oscillated in reverse directions along that line. ii: For a delay of 4 ms, two oscillations were allowed until TH disrupted the waves. iii: By increasing delay (6 ms), infinite oscillations are sustained in opposite directions after the initial trigger. (**f**) Space-time projections of traveling waves for two delay parameters used in ii and iii. (**g**) Schematic showing how the TH inhibition can be used to break the CX wave. A broken wave tends to curl around a tip. (**h**) An illustration of generating a rotating cortical wave, which emerged when the TH inhibition was reduced at time t0. (**i**) Space-time projection of the wave shown in panel h.

Having shown that the randomly selected unconnected TH-CX nodes produced spontaneous traveling wave patterns, we further investigated whether and how the change of TH-CX connectivity could predict the specific propagating wave type, allowing potential control over the ensuing wave pattern. Specifically, we used the same setup as in Fig. 4a (i.e., nonzero delay and fully connected TH-CX), where the cortical wave had a particular firing range (the cortical firing field, dashed box in Fig. 5a), i.e. a particular spatial range it could traverse before being extinguished by the thalamic inhibition.

We considered four distinct scenarios depending on the location and number of the triggered nodes. In the first scenario, in which an unconnected node was chosen within the range of cortical firing field—this node fired for a longer duration in time because it was not affected by TH inhibition (shown by a black dashed circle in Fig. 5a). However, because of the surrounding cortical firing refractoriness, the firing did not generate a cortical wave that propagated spatially. In the second scenario, in which the unconnected node was at the corner (edge) of the cortical firing field (Fig. 5b), a cortical wave emerged in space since the cortical refractoriness was absent outside the field. The cortical wave initiated outside the dashed box, and then became unconstrained once the refractoriness terminated (Supplementary Video S9). In the third scenario, two unconnected points were selected to create two planar waves in opposing directions (Fig. 5c). Therefore, for a fixed set of connectivity and delay parameter, the traveling wave patterns (e.g. direction, area, and speed) could be predicted by our simulation model (Fig. 5d). On the other hand, when the unconnected node was outside the cortical firing field, no wave pattern could be produced.

In the fourth scenario, when the unconnected nodes were chosen in the form of a straight line (Fig. 5e, subpanel *i*), the resulting cortical wave oscillated in reverse modes along that line (delay of 4 ms for $t=1$–10 ms, a temporary threshold block was used to initiate a unidirectional wave). Under the 4 ms delay condition, two oscillations could be sustained until the thalamic inhibition disrupted the cortical wave ($t=22$–60 ms) (Supplementary Video S10). On the other hand, if the thalamocortical delay was increased from 4 ms to 6 ms (from $t=22$ ms onwards) during the simulation (Fig. 5e, subpanel *iii*), then cortical wave oscillations were sustained indefinitely in opposite directions (Supplementary Video S11). A time-course of these neuronal oscillations is illustrated in Fig. 5f, along with a 1D space-time representation. Note, that observing these oscillations in time, without a spatial readout, was insufficient to assess the reverse directions.

An interesting observation of spontaneous traveling wave patterns in our 2D thalamocortical model was the rotating wave. Figure 5g illustrates the schematic of one of many possible ways of producing a rotating wave, in which the thalamic inhibition was used to break a planar wave, followed by a reduced inhibition level. A broken planar wave tends to curl around its tip to create a rotating wave^35,45,46^. If the thalamic inhibition was not reduced, the spiral would not have sufficient time to evolve (Supplementary Video S12). Figure 5h shows the implementation of this method where the level of thalamic inhibition was reduced by ten-fold at the moment of $t=28$ ms. As a consequence of reduced thalamic inhibition, a rotating cortical wave emerged and sustained indefinitely (Supplementary Video S13). The 1D space-time projection of spiral cortical waves is illustrated in Fig. 5i. As seen in our simulations, in this case, even a 1D space-time representation was insufficient to comprehend the underlying spiral wave fully, and only the 2D traveling wave representation could reveal the complete picture.

To study the evoked traveling wave, we further incorporated an external input to the 2D network that generated the spiral wave, which caused the spiral to enhance (or reduce) depending on the excitatory (or inhibitory) nature of the input Supplementary Video S14 and Supplementary Video S15). Together, the results suggest that based on the thalamocortical connectivity and transmission delays, one can qualitatively predict the characteristics of the spatiotemporal patterns that may ensue as a result of perturbations. Finally, to verify that these results were not solely due to the size of the simulation domain but rather to the extent of cortical-fire field relative to the size of the neural array, we increased the array size from 60$\,\times\,$60 to 80$\,\times\,$80 and re-ran the computer simulations. Different wave patterns were still obtained through the unconnected points at the edge of the firing field (Supplemental Videos S16).

### Thalamic and cortical excitation/inhibition imbalance alters traveling wave frequencies and speeds

Excitation/inhibition balance in neural circuits is critical for brain functions, and E/I imbalance may induce dysfunctional physiology such as epilepsy and seizures^47^. To investigate the effect of E/I balance on traveling wave characteristics, we focused our attention on a clustered cortical group, in which cortical neurons were nearly fully connected (with 80-90% intracortical connectivity), in our three-layer network model.

**Figure 6:**
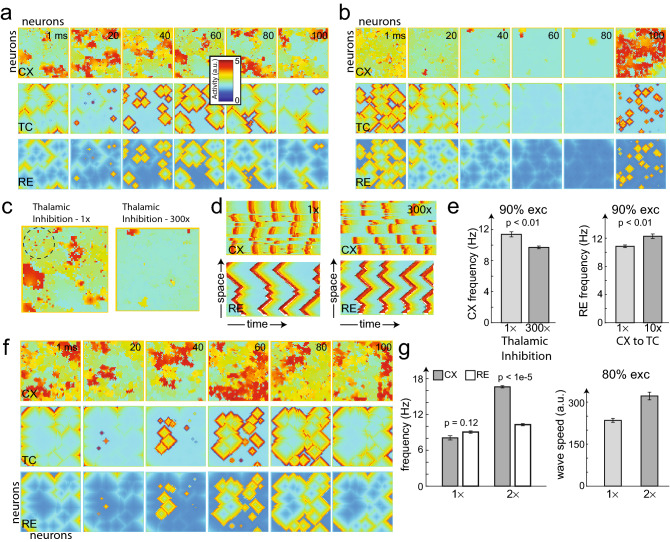
Cortical and thalamic E/I balance alters traveling wave speed and frequency. (**a**) The spatiotemporal activity produced by a closed-loop 2D thalamocortical network (10% TH$\rightarrow$CX connectivity, fully connected CX with 90% excitatory neurons). Each panel shows the 60$\,\times\,$60 neuron array layout and colors denote activity levels. (**b**) Spatiotemporal activity obtained with the same setting as a, but with RE inhibition increased. (**c**) Repeated cortical cell firings occurred due to TC excitatory inputs (dots in the dashed black circle). These dots propagated as a traveling wave. Triggering dots reduced/absent when RE inhibition increased. (**d**) 1D projections of waves from the two scenarios. (**e**) (left) Comparison of the cortical oscillation frequency between two levels of RE inhibition ($1\times$ vs. $300\times$). (right) Comparison of the thalamic oscillation frequency between two levels of CX$\rightarrow$TC excitation ($1\times$ vs. $10\times$). All error bars represent SEM. (**f**) Traveling waves induced by increased CX excitatory weights (10% TH$\rightarrow$CX connectivity and 99% intracortical connectivity with 80% excitatory neurons). As seen in space-time projections, wave activity was significantly increased (in comparison with Fig. 1d). (**g**) Cortical wave frequency and speed increased as the CX excitatory weights were multiplied by two folds (*p* values obtained from five simulations, Student’s* t* test).

First, we examined the impact of E/I imbalance by changing the RE inhibition on the cortex. To help illustrate this point, we assumed that the cortex contained 90% excitatory neurons. In a closed-loop setting, we compared the 2D thalamic and cortical traveling wave dynamics, between regular (Fig. 6a) and increased (Fig. 6b) RE inhibition. In these cases, the RE and TC neurons competed to trigger the cortical wave activity. In the presence of lower RE inhibition, TC excitation dominated, triggering cortical neuronal firing (Fig. 6c, left, red dots in a black circle indicate nascent triggers). These dots ultimately propagated to form cortical waves. However, with increased RE inhibition, the effect of TC excitation decreased (Fig. 6c, right—the dots are absent), resulting in fewer cortical traveling waves, or lower frequency (Supplementary Video S17). This can be further appreciated by noticing the large gap in time between successive space oscillations in Fig. 6b, contrasting that with the oscillations in Fig. 6a. Also, comparing the number of striped firing patterns in 1D projections (Fig. 6d), we observed a decrease in traveling wave frequency in the cortex induced by increased RE inhibition. Therefore, increased RE inhibition lowered the cortical wave frequency, while reduced $\mathrm {CX}\rightarrow \mathrm {TC}$ weights increased the thalamic wave frequency (Fig. 6e). In contrast, the change in cortical wave speed was insignificant, suggesting that the cortical wave was relatively stable regardless of the level of thalamic inhibition.

Finally, we examined the effect of imbalance in cortical excitation on traveling waves, by increasing the excitatory intracortical weights by two-fold. As a result, we observed a dramatic change in traveling wave patterns (Fig. 6f, Supplementary Video S18), as well as a significant increase in both cortical wave frequency and wave speed (Fig. 6g). With a 4:1 excitatory to inhibitory neuron ratio, the excitable parameters were assumed such that the thalamus and cortex were synchronized in frequency. However, as we increased the cortical excitation two-fold, a difference between the RE and CX frequencies emerged (Fig. 6g). Together, the results suggest that cortical E/I balance affects the traveling wave frequency and speed—a result in line with a previous 1D model that showed that the traveling wave speed increases (logarithmically) with the synaptic coupling strength^25^.

## Discussion

We developed a 2D topographic network of the closed-loop thalamocortical system that produces a broad class of spontaneous or evoked spatiotemporal wave patterns and oscillations in the cortex and thalamus. This architecture was able to sustain smooth waves in the cortex and lurching waves in the thalamus simultaneously. Our computer simulations showed that the propagating wave patterns are influenced by many factors such as intracortical and thalamocortical connectivity, cortical E/I imbalance, thalamic inhibition, and thalamocortical or corticothalamic delay. Specifically, altering these parameters allowed us to change traveling wave speeds, directions, patterns, and oscillation frequencies, suggesting a simple computational mechanism for the genesis of the diverse wave patterns observed in the brain. Thus, it is possible to replicate the experimentally observed wave characteristics through simple modifications of connectivity or synaptic weights, without going into neural-field continuum approximations^27^. Furthermore, we showed that the 2D traveling waves may display unique characteristics that are indiscernible in 1D projections or in vitro (isolated) conditions.

### Wave oscillations, speed, and frequency

Different oscillation frequencies are known to reflect various brain states. Slow alpha oscillations are observed during sleep and anesthesia^8^, whereas beta/gamma oscillations are typical of attention and memory tasks^48^. These oscillations are mostly thought to occur from bifurcations in the governing voltage equation, which render the equilibrium unstable creating oscillatory dynamics^40^ or from stochastic inputs to neurons. Our computer simulations suggest an alternative where, using the closed-loop architecture, traveling wave oscillations in space-time can be generated and sustained deterministically without having an explicit bifurcation through parameter variations or relying on additional stochastic inputs to neurons. This occurred owing to the feedback and feedforward connections, where a cortical wave triggered a slower thalamic wave, which, in turn, triggered another wave in the cortex and so on. This time-scale separation between the thalamic and cortical wave speeds was important, as this allowed the refractory period of the cortical neurons to end before the thalamic wave had been extinguished—enabling the next cortical wave trigger. Our result closely ties in with one published study, which showed cortical alpha oscillations during predictive coding^12^. They also suggest that natural oscillations can be generated and sustained owing to the inherent hierarchical structure of the cortical layers. Our study further supports the possibility of simultaneously sustaining different types of oscillations (lurching waves and smooth waves) in different layers. Cortical oscillations are known to host strong feedback and feedforward connections, both between the laminar layers of the cortex and with the deep thalamic layers^49^. These connections may create ample opportunities for generating closed-loop oscillations—allowing smooth and staggered waves to sustain in different layers simultaneously. The speed of cortical traveling waves may have a wide range, ranging from 0.1–0.8 m/s for mesoscopic waves, to 1–10 m/s for macroscopic waves^11,50^. Multiple factors, including neural recording techniques and spatial coverage, may contribute to the diverse values of traveling wave speed reported in the literature. Our computer simulations predict that the cortical wave speed can be influenced by excitatory cortical synaptic connections. We further demonstrate that increased neural oscillation frequencies can be associated with faster propagating waves. This is because, in the closed-loop architecture, a lower system threshold not only increases activty in one layer, but also translates to increased inputs to all connected layers. Consequently, not only is the wave speed increased, but the overall system frequency also rises.

### Information propagation

Traveling waves in the brain are believed to play important functional roles including memory consolidation, processing of dynamic visual stimuli, sensorimotor integration, and multisensory discrimination and gating^4,11,37^. One of the speculative roles of macroscopic traveling waves is to propagate and coordinate information across multiple brain regions in space and time^2,51^. Recent experimental findings have shown that thalamic traveling waves may be critical for the development of cortical representations from different sensory modalities^52^. Our results support the hypothesis that a potential role of traveling waves is to enable information being transferred between different cortical areas or to generate spatial coherence^53^. In this case, we show that spatial coherence may also be obtained in disconnected cortical areas through thalamic traveling waves. It has been shown that divergent thalamic inputs to the cortex result in a synchronization of the cortical activity^32,36^. Our simulation ties in with these studies while additionally suggesting that information transfer across unconnected regions of the cortex can also occur using global-scale thalamic connections.

### Pattern formation using conduction delay and connectivity differences

Transmission delay between adjacent neuronal connections is known to cause bifurcations resulting in altered dynamics^25,44^. The long-ranging thalamic inhibition relies upon divergent thalamic connections to the cortex^32^, but there are significant axonal conduction delays. Our computer simulations confirmed that the thalamocortical delay can produce a wide range of emergent spontaneous traveling wave patterns, through similar mechanisms of pattern formation using long-range inhibition as discussed in the introduction while avoiding neural-field approximations^27,29^. Specifically, the “race” between the cortical wave speed and the delayed, long-ranging thalamic inhibition (creating a finite cortical firing field)—corresponds to the time and space-scale separation needed for pattern formation^35^. The exact wave type generated depended on the connectivity with respect to the firing field, the spatial extent of the connections and the delay. The delay parameter elicited a biphasic response, suggeasting that an optimum delay exists for a particular neural field size to generate the maximum number and duration of specific wave patterns. These patterns can be controlled by perturbing network connectivity, to alter wave speed, direction, and frequencies. Thus, it is possible to acutely modulate the wave pattern observed. In experiments, to fully comprehend the differences in wave patterns, however, a readout from 2D spatial recordings is necessary. Throughout our computer simulations, we used a thalamocortical delay to generate traveling waves. As evidenced from the literature^54,55^, the corticothalamic delay is more prominent compared to the thalamocortical delay. As we demonstrated in Supplementary Fig. S3, an asymmetric corticothalamic delay also produced qualitatively similar cortical traveling wave alterations as the TH-CX connections were changed. In a closed-loop setting, the exact location of the delay along the neural pathway (feedforward vs. feedback) does not change the logic behind wave pattern alterations, since the pattern formation theory necessitates only a long-range antagonist that is delayed in time when compared to the local activity^56^ .

*Excitation/inhibition imbalance:* Through numerical simulations, large-scale computational models may provide insights into the spatiotemporal dynamics of the thalamocortical network at a pathological brain state. The cortical or thalamic E/I imbalance is an important factor that contributes to epilepsy and seizures^47^. Our results suggest that in a clustered cortical network, increasing the E/I ratio drastically increases the traveling wave speed and overall neuronal excitability, a phenomenon commonly observed in the pathological brain. For instance, traveling waves have been observed during epileptic seizures^53,57,58^, but a complete understanding of their origin remains unclear. One potential mechanism of absence seizure (one kind of primary generalized seizure) is thalamic dysfunction^59–61^. Another plausible mechanism of recurrent seizure is E/I imbalance induced by stronger cortical excitation, which further causes the neuronal network to reach hyperexcitability^62^. Our computer simulations suggest that the closed-loop thalamocortical system is important for cortical wave propagation, and that the input of excitatory TC cells is necessary to maintain high oscillation frequencies, and that subduing TC input through thalamic inhibition can significantly reduce thalamocortical oscillations. This is consistent with experimental results in a rat model that suggested the requirement of the thalamic input to maintain cortical seizure oscillations, and that optogenetic inhibition of TC cell activity disrupts seizure oscillations^63^. Therefore, the dynamic properties of spatiotemporal traveling waves, such as the wave speed, direction, and duration, may provide a diagnostic window to examine pathological brain functions.

### Difference from other models in literature

Our computational model differs from other models in the literature developed to account for traveling waves in the brain. The majority of these are based on the integro-differential equations^64^. While these equations capture the basic wave dynamics, they approximate the nonlinear positive feedback with a sigmoidal threshold response, thereby neglecting important phenomenon that our model can capture, such bifurcations and bistabliity. Another common method is to use the neural-field approximation to generate wave patterns, replacing synaptic operators with double-spatial derivatives, akin to diffusion^26,27^. In contrast, our computational model is capable of generating different patterns using connectivity differences without an explicit diffusion approximation. Similar to the integro-differential equations, our model is also phenomenological as it neglects some biophysical details (such as the voltage-dependent synaptic conductance) and replaces adjacent connections through a direct input term. We derive the basic structure of our model from the Izhikevich type neurons^38^ and validate our parameter setup by recreating the lurching wave of the thalamus, and the smooth waves of the cortex. We use a threshold approximation similar to Type-1 neurons^38^, where the network connectivity and synaptic inputs essentially alter the neuron’s proximity to the firing threshold. This is an oversimplifying assumption used to reduce the computation time because numerical simulations of spatiotemporal activity of a large-scale network based on biophysically-detailed equations can be computationally cumbersome. Although we have used a phenomenological computational model, our wave propagation findings should be generalizable to other more biophysically-based neuronal models. Many studies in the literature have used approximations of biophysical details to analyze spatiotemporal patterns^27^ since it is more difficult to gain insight from models with high-dimensional parameter space. Because of these simplifications, the exact values of the parameters and their corresponding output wave speed/area values are not as important as is the relative change when the system parameters are altered. Several 2D models have been developed for cortical structures^27,45,65–67^, but very few have focused on the thalamocortical structure. To date, the available 1D models for thalamocortical systems have not explicitly modeled the network connectivity topography (i.e. the clustered intracortical connectivity) or did not jointly model transmission delay and thalamic inhibition^25^. Furthermore, as shown in our simulations, the 1D projection has limited capability of characterizing traveling wave patterns or properties.

### Model limitations and future directions

Our model does not account for the detailed laminar structure of the cortex, nor does it account for the intra-laminar connections^24,32,68^. Incorporating more connectivity constraints within the cortical layers would further add anatomical details to the neural circuitry^69^. Our numerical simulations of the 2D thalamocortical network were still on a relatively small scale, therefore, some neurobiological details could not be modeled fully. For instance, a separate treatment of thalamocortical and corticothalamic feedback may account for another level of complexity^70^. Additionally, the bursting behavior of RE cells at rest has been omitted in our current model, and only tonic spiking has been studied. The introduction of bursting may have interesting effects on the ensuing wave—a topic that needs further exploration. Lateral thalamic inhibition was also taken from the RE layer for simplicity. This thalamic inhibition was also studied ignoring the effect of the thalamic lurching wave, which would add further diversity to the wave patterns. Finally, we have only considered local intra-cellular connections and ignored long-range axonal connections within a layer. These short-range connections, for example, resulted in thalamic waves being at a 45-degree shape in our numerical simulations. Future work will be required to investigate these issues in greater detail.

While state-of-the-art electrophysiological recordings allow recording of a large number of cortical neurons in 2D or 3D^71–74^, simultaneous recordings of a large number of cortical and thalamic neurons based on multi-site multielectrode arrays remains a technical challenge due to the size and anatomy of the thalamus. The depth of thalamic structure also brings challenges in optical imaging for large-scale thalamic cells. These factors create difficulty for in vivo experimental verification of cortical and thalamic traveling waves. However, as future neural recording technologies are improved^75^, our model predictions motivate the need for recording cortical and thalamic traveling waves simultaneously. Our model prediction overall provides a new testable hypothesis that the traveling wave patterns continually observed on the cortex can be the result of specific connectivity differences and can potentially be controlled to affect brain functions.

## Methods

### Network architecture, neuron dynamics, and threshold analysis

We developed a three-layer thalamocortical system, each layer consisting of a 2D lattice of neurons, modeling two layers of the thalamus and one cortical layer interconnected through feedback and feedforward connections (Fig. 1a,b). Every neuron is described by three differential equations. Two describe the voltage (*v*), and gating ($\eta$) terms (Fig. 1c) which generate excitable behavior^38^, along with a synapse output equation. The first two states approximate a spiking neuron by assuming instantaneous activation of the sodium current (fast increase of voltage term), with a slower potassium inactivation (slow gating variable). This gating variable had voltage-dependent steady-state and time-constant terms which were approximated in our model equations to ensure that the dynamics matched that of the excitability model^35,38^, i.e. recreated spiking and oscillations for one neuron. This can be better visualized in the phase plane (Supplementary Fig. S1a). Each point in the phase plane describes how the system states ($v,\eta$) would evolve over time if the system were to start at that state. The two nullclines are points for which one of the two states does not change. In our model of the neurons, the curve with the inverted ‘N-shape’ is the voltage nullcline on which *v* does not change and the straight line is the nullcline for the gating variable does not change. The intersection of the two nullclines, denoted ‘m’, is the equilibrium. In the situation illustrated in Supplementary Fig. S1a, where the equilibrium is to the left of the minimum point of the *v*-nullcline (denoted ‘p’), the equilibrium is stable. Changing either nullcline alters the equilibrium. For example, if the *v*-nullcline is raised (e.g. through the input *r* in Supplementary Fig. S1a), then the equilibrium point moves closer to point ‘p’—which is a bifurcation point. Once the two points meet, the equilibrium loses its stability, at which point it begins self-sustaining oscillations. For this reason, we refer to the distance between the points ‘p’ and ‘m’ as the activation “threshold” of the system. The stability of the system can be determined from the eigenvalue plot in Supplementary Fig. S1b^76^. Here, the threshold was altered by lowering the slope of the gating variable nullcline (Supplementary Fig. S1c, top, red arrow).

Each module, thus, had an activation threshold that could be controlled through the system parameters (Supplementary Fig. S1a). If this input was sufficient enough to displace the state beyond the activation threshold—the state would undergo a large excursion in phase-space (shown through grey arrows in Supplementary Fig. S1a)—resulting in a spike of activity. Overall, the form of our differential equations representing a neuron matched the phase-plane architecture and dynamics of established neural two-state models^38^.

Following established neuronal classification criteria^38,77^ we assumed that thalamic neurons belong to Type-3, which generate only a single spike following a step input. This was ensured by adjusting the model parameters such that the phase-plane structure (Supplementary Fig. S1c, top) was similar to that shown in earlier studies^38^ (same phase plane as Supplementary Fig. S1a). This allowed neurons to exhibit oscillations whenever the input was sufficiently large, but the oscillation frequency remained relatively constant for a wide range of input strengths. This is illustrated in Supplementary Fig. S1d, where different input strengths were applied to the neuron after the bifurcation had occurred. This neuron also exhibited a rebound spike following an inhibitory synaptic input. We also assumed that cortical neurons belong to Type-1, which exhibit a wide range of oscillation frequencies for different input strengths and could reach an arbitrary slow frequency (illustrated in Supplementary Fig. S1d). This was also ensured by adjusting the model parameters such that the phase-plane structure (Supplementary Fig. S1c, bottom) was similar to that of earlier studies^38^, with its corresponding stability plot (Supplementary Fig. S1e). Here, the threshold of the cortical neuron was altered by lowering the position of the gating nullcline (Supplementary Fig. S1c, bottom, red arrow).

A neuron spike would generate a synaptic output (Supplementary Fig. S1d). Every neuron had a synapse equation that fed to other neurons. A neuron could be excitatory or inhibitory depending on the sign of its synapse—i.e. based on if it excited/inhibited the post-synaptic neurons respectively (Fig. 1d). Lateral connections of these neuron modules created a neural sheet. Detailed parameters are presented in Tables 1 and 2. The cortical neural sheet was modelled through 4-way connectivity, i.e. each CX neuron could be connected to its adjacent 4 neurons. Cortical neurons were surmised at 80% excitatory and 20% inhibitory neurons^33^. Intra-cortical connections, reported to be around 25%^41^, were varied from uniformly sparse to locally clustered network structures. This was done through randomly removing existing connections till the overall connectivity reached the desired value. In the uniformly sparse case, connections were removed randomly throughout the sheet. In a locally clustered setup, the 4-way connections were left intact for a particular group of neurons (the cluster), while connections outside the group were removed such that the total connectivity remained the same.

The cortex and thalamus were connected in closed loop where a sum of excitatory thalamocortical (TC) and inhibitory reticular (RE) activity influenced the cortical activity, while the cortex projected to both RE and TC through excitatory connections^39^ (Fig. 1e). The corticothalamic feedback connectivity between the cortex and the thalamus was set at 1%, i.e. 1% of randomly chosen cortical neurons fed back to thalamic ones, while thalamocortical connections were set similarly at 10% connectivity. RE and TC layers were interconnected through alternate reciprocal connections^18^. The lateral or intra-RE connections were not modeled here. The schematic connections within the thalamocortical network are illustrated in Fig. 1e.

### Neuron equations and model setup

The model equations used for the different neurons are described below.

*Thalamic relay cells (TC).* These neurons are described by the voltage term, $T_v$, gating variable, $T_\eta$, and synapse, $T_s$, obeying the following equations:

1a $$\begin{aligned} \frac{\mathrm {d}T_v}{\mathrm {d}t}&=-(a_1+a_2T_\eta )T_v+\frac{a_3T_v^2}{a_4+T_v^2}+a_5+T_{\mathrm {inp}}\end{aligned}$$

1b $$\begin{aligned} \frac{\mathrm {d}T_\eta }{\mathrm {d}t}&=-c_1T_\eta +c_2T_v\end{aligned}$$

1c $$\begin{aligned} \frac{\mathrm {d}T_s}{\mathrm {d}t}&= \frac{\frac{1}{2} \left( 1+\tanh { \left( 120 \left( T_v-\frac{1}{10}\right) \right) } \right) -T_s}{t_1 \left( s_1-{\frac{1}{2}} \Bigl ( 1+\tanh { \bigl (120 (T_v-\frac{1}{10}) \bigr )} \Bigr ) \right) } \end{aligned}$$

The coefficients $a_1,\ldots ,a_5$ determine the shape of the voltage nullcline giving it an inverted-N-shape typical of excitable systems. In contrast, the coefficients $c_1$ and $c_2$ regulate the slower gating dynamics and result in a linear nullcline. The thalamic cells were set up as Type-3 neurons^38^ (Supplementary Fig. S1c,d). The input to the neuron raises the voltage nullcline allowing the activation threshold to be crossed to generate firings (similar to *r* term in Supplementary Fig. S1a). This input to the TC layer is given by:

2 $$\begin{aligned} T_{\mathrm {inp}}= -w_{\mathrm {RE-TC}} C_{\mathrm {TH}}R_s\left( T_v+i_p\right) + w_{\mathrm {CX-TC}} {M_{\mathrm {sparse}}}C_sT_v. \end{aligned}$$

The first term consists of an inhibitory input from the reticular layer (RE) synapse, $R_s$. The term $i_p$ is a constant used to modulate the inhibition level. The connectivity matrix, $C_{\mathrm {TH}}$, connects every TC element to the surrounding four nearest neighboring RE neurons. The second term represented an excitatory input from the cortical synapse ($C_s$), multiplied by a sparsity matrix, denoted $M_{\mathrm {sparse}}$. The terms $w_{\mathrm {RE}\text{- }\mathrm {TC}}$ and $w_{\mathrm {CX}\text{- }\mathrm {TC}}$ denote the respective weights of the RE and CX synapses on the TC neurons.

*Thalamic reticular nuclei (RE) layer.* These neurons are governed by equations similar to the TC neurons (Eq. 1) with a voltage term, $R_v$, gating variable, $R_\eta$, and synapse, $R_s$. The parameters specifying the dynamics of the RE neurons were chosen to be the same as the TC case for Type-3 neurons. The input to the RE layer equals:

3 $$\begin{aligned} R_{\mathrm {inp}}=+w_{\mathrm {TC}\text{- }\mathrm {RE}}T_sR_v+w_{\mathrm {CX}\text{- }\mathrm {RE}}M_{\mathrm {sparse}}C_sR_v. \end{aligned}$$

Note that both terms are excitatory, the first from the TC synapse ($T_s$) and the second from the CX synapse ($C_s$), multiplied by the thalamocortical $\mathrm {CX}\rightarrow \mathrm {RE}$ sparsity matrix $M_{\mathrm {sparse}}$. Furthermore, the net input to each RE neuron was saturated at a minimum to prevent numerical instabilities. No intra-RE connections were assumed.

In the simulations of Figs. 4 and 5, we introduced a corticothalamic delay in the model by using a delayed CX input ($C_{\mathrm {delay}}$ instead of $C_s$) to the cortex. To avoid using delay-differential equations explicitly, we implemented this delay by taking the synapse from the CX neuron ($C_s$) and passing it to a series of “fictitious” neurons consisting of three reactions, similar to those of Type-3 neurons and generating a post-inhibitory rebound spike (Supplementary Fig. S4). Each of these delay circuits induced a 2 ms delay due to the wait time before the rebound spike. Thus, three such elements caused a total tramsmission delay of 6 ms.

*Cortex (CX) layer.* These neurons have a voltage term, $C_v$, gating variable, $C_\eta$, and synapse, $C_s$ whose dynamics are governed by the following equations:

4a $$\begin{aligned} \frac{\mathrm {d}C_v}{\mathrm {d}t}&=-(a_1+a_2C_\eta )C_v+\frac{a_3C_v^2}{a_4+C_v^2}+a_5+C_{\mathrm {inp}}\end{aligned}$$

4b $$\begin{aligned} \frac{\mathrm {d}C_\eta }{\mathrm {d}t}&=-d_1C_\eta +d_2\frac{C_v^3}{d_4+C_v^3}+d_4 \end{aligned}$$

4c $$\begin{aligned} \frac{\mathrm {d}C_s}{\mathrm {d}t}&= \frac{\frac{1}{2} \left( 1+\tanh { \left( 120 \left( C_v-\frac{1}{10}\right) \right) } \right) -C_s}{t_1 \left( s_1-{\frac{1}{2}} \Bigl ( 1+\tanh { \bigl (120 (C_v-\frac{1}{10}) \bigr )} \Bigr ) \right) }. \end{aligned}$$

The cortical inhibitory neurons were set up as Type-1 neurons (Supplementary Fig. S1c,d), which do not exhibit inhibitory postsynaptic rebound. Instead, they have saddle-node bifurcations that allow different spiking frequencies for different input strengths. The input to the neuron raises the voltage nullcline allowing the activation threshold to be crossed to generate firings (similar to *r* term in Supplementary Fig. S1a). This input, $C_{\mathrm {inp}}$, consists of the sum of six components: $C_{\mathrm {inp}}={C_{\mathrm {inp}}}_1+\cdots +{C_{\mathrm {inp}}}_6$, where each is given by:

1. Excitatory input from TC synapses ($T_s$) weighted by $w_{\mathrm {TC}\text{- }\mathrm {CX}}$ and connectivity matrix $W_{\mathrm {sparse}}$:
  5a $$\begin{aligned} {C_{\mathrm {inp}}}_1&=+w_{\mathrm {TC}\text{- }\mathrm {CX}}W_{\mathrm {sparse}}T_sC_v. \end{aligned}$$
2. Inhibitory inputs from RE synapses ($R_s$) weighted by $w_{\mathrm {RE}\text{- }\mathrm {CX}}$ and connectivity matrix $W_{\mathrm {sparse}}$;
  5b $$\begin{aligned} {C_{\mathrm {inp}}}_2&=-w_{\mathrm {RE}\text{- }\mathrm {CX}}W_{\mathrm {sparse}}R_s(C_v+i_p). \end{aligned}$$
3. Excitatory inputs from neighboring CX synapses ($C_s$) weighted by $w_E$, connectivity matrix $X_{\mathrm {sparse}}$, and the total number of neurons (*N*);
  5c $$\begin{aligned} {C_{\mathrm {inp}}}_3&=+w_E\frac{k_n}{\sqrt{N}}X_{\mathrm {sparse}}C_s. \end{aligned}$$
4. Inhibitory inputs from neighboring CX neurons with synapse ($C_s$) weighted by $w_I$, the connectivity matrix $X_{\mathrm {sparse}}$ and the total number of neurons (*N*);
  5d $$\begin{aligned} {C_{\mathrm {inp}}}_4&=-w_I\frac{k_n}{\sqrt{N}}X_{\mathrm {sparse}}C_s. \end{aligned}$$
5. Inhibitory inputs from the thalamic layer synapses ($L_s$) (in the reduced TH-CX model) multiplied by weights $w_{\mathrm {TH}\text{- }\mathrm {CX}}$ and connectivity matrix $W_{\mathrm {sparse-TH}}$;
  5e $$\begin{aligned} {C_{\mathrm {inp}}}_5&=-w_{\mathrm {TH}\text{- }\mathrm {CX}}W_{\mathrm {sparse-TH}}L_s(C_v+i_p). \end{aligned}$$
6. A stochastic, Gaussian noise input with mean $\mu$ and variance $\sigma$:
  5f $$\begin{aligned} {C_{\mathrm {inp}}}_6&=+\mathcal {N}(\mu ,\sigma ). \end{aligned}$$

When considering the thalamocortical delay, the term $R_s$ in ${C_{\mathrm {inp}}}_2$ was replaced by $R_{\mathrm {delay}}$. This was achieved by passing the RE synapse, $R_s$, through multiple post-inhibitory rebound spikes before it reached the cortex, as in the cortical delay scheme mentioned above. We assumed that the intracortical connectivity was sparse (characterized by a binary and symmetric matrix $X_{\mathrm {sparse}}$, which incorporated the effect of clustering or rearrangement of cortical cell connections. We also assumed that the excitatory-to-inhibitory neuron ratio in the cortex was 4:1.

*Thalamic inhibitory layer for reduced model (TH).* This layer was only used with the CX layer in the reduced model case of Figs. 4 and 5. It uses the same equations as the TC neurons (Eq. 1), with a voltage term, $L_v$, gating variable, $L_\eta$ , synapse, $L_s$, and input term:

6 $$\begin{aligned} L_{\mathrm {inp}}=+w_{TH}C_{\mathrm {TH}}L_vL_s+w_{\mathrm {CX}\text{- }\mathrm {TH}}W_{\mathrm {sparse-TH}}C_sL_v\end{aligned}$$

This layer received inputs from adjacent thalamic excitation (first term), and cortical inputs (second term), while it exerted inhibitory inputs on the cortex. The CX layer remained the same with 100% excitatory neurons (${C_{\mathrm {inp}}}={C_{\mathrm {inp}}}_3+{C_{\mathrm {inp}}}_5+{C_{\mathrm {inp}}}_6$), and was completely connected.

### Numerical simulation details and traveling speed characterization

The neural equations were solved numerically using the SDE toolbox of MATLAB (MathWorks)^78^ , using a 2D array for a neural layer with absorbing (high-threshold) boundary conditions. Kymographs were calculated from the 2D activity obtained from the model species through line scans in time. In our model, each layer consisted of 3,600 neurons (array of 60$\,\times\,$60), the computer simulation time for the proposed three-layer network was $\,\sim \,$600 s for 400 ms duration. Statistics were done by varying the (feedback, feedforward and intracortical) network connectivity and the arrangement of excitatory neurons in the cortex. Student’s t-test was used to calculate statistical significance.

To measure the traveling wave speed, a custom MATLAB script was used. Briefly, the wavefront was segmented at subsequent frames of videos. Based on these segmentations, the number of patches and averaged area were computed. To measure the averaged wave speed, the distance from each pixel on the boundary of a wavefront in frame $n+1$ to the closest edge of a wave in frame *n* was computed. Our computed wave speed was in an arbitrary unit (a.u.). To put that in a perspective, if we record a 60$\,\times\,$60 array of neuronal activity from a 2D multielectrode array (MEA) of $\,\sim \,$24$\,\times\,$24 mm^2^ , then each frame of the wave videos reflects the 2-ms temporal resolution such that the simulated wave speed is $\,\sim \,$100–300 cm/s. Usual LFP recording arrays are of the order of 3-4 mm in dimension, but a broader neural sheet is studied for clarity of wave structure. A couple of zoomed-in views (10$\,\times\,$10 array) are also provided for comparisons with recorded data.

## Supplementary Information

Supplementary Information 1.

Supplementary Video 1.

Supplementary Video 2.

Supplementary Video 3.

Supplementary Video 4.

Supplementary Video 5.

Supplementary Video 6.

Supplementary Video 7.

Supplementary Video 8.

Supplementary Video 9.

Supplementary Video 10.

Supplementary Video 11.

Supplementary Video 12.

Supplementary Video 13.

Supplementary Video 14.

Supplementary Video 15.

Supplementary Video 16.

Supplementary Video 17.

Supplementary Video 18.
