# Supplementary material for: The impact of a closed-loop thalamocortical model on the spatiotemporal dynamics of cortical and thalamic traveling waves
Source: Sci Rep. 2021 Jul 13;11:14359. doi: 10.1038/s41598-021-93618-6 (PMC8277909; doi:10.1038/s41598-021-93618-6)
Supplement: Supplementary file 1 — Supplementary Information 1. [file 41598_2021_93618_MOESM1_ESM.pdf]

The impact of a closed-loop thalamocortical model on the spatiotemporal dynamics of cortical and thalamic traveling waves

Supplemental Information

Sayak Bhattacharya, Matthieu B. L. Cauchois, Pablo A. Iglesias, Zhe Sage Chen

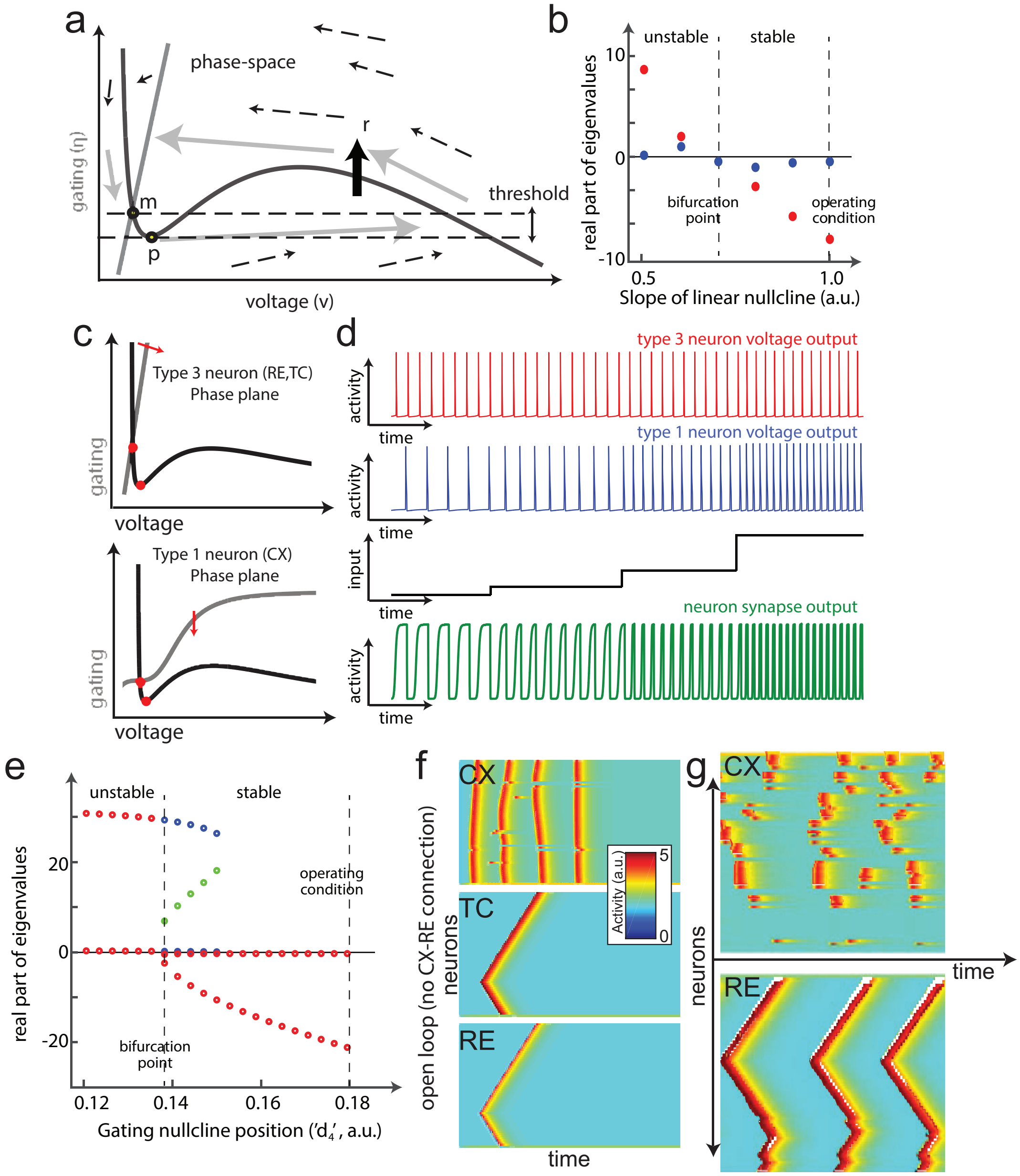

Supplementary Figure S1. (a) Phase-space representation of the excitable module. The curves denote the nullclines, and the arrows show the trajectories that the state would take at that position. The larger grey arrows show the trajectory during a spike response. Symbols 'm' and 'p' denote the equilibrium and bifurcation points respectively, with the gap between them indicating the activation threshold. The black vertical arrow 'r' shows how an external input would lower the threshold of the system. (b) Bifurcation diagram of the two-state system of panel 'a' showing the eigenvalues (real part of two eigenvalues red and blue) of the system as the activation threshold is altered by changing the slope of the gating nullcline (arrow in panel 'c' top). Positive eigenvalues indicate an unstable state (bifurcation). (c) Illustrations of phase plane for Type-1 and Type-3 neurons (Izhikevich's classification) used in our computer simulations. Arrows indicate how the activation threshold can be altered, thus reducing the gap between the equilibrium and the bifurcation point (two red dots). (d) Spike responses of the two types of neurons and synaptic output to increasing input strengths. These inputs pushed the neurons beyond the bifurcation point, generating oscillations. The oscillation frequency of the Type-1 neuron varied noticeably with input strength. (e) Bifurcation diagram of the two-state system of the Type-1 cortical neuron, showing eigenvalues (real part) of the system as the activation threshold is altered by moving the gating nullcline in phase space (arrow in panel 'c' bottom). Positive eigenvalues indicate an unstable state (bifurcation). Two eigenvalues are consistent for one equilibrium (shown in red). Near the bifurcation point of this equilibrium, two new equilibria emerge (green and blue) which are unstable. (f) Illustration of open-loop traveling wave dynamics (shown in the form of space-time projections) of the thalamocortical system when both the thalamus and cortex were manually triggered at a point initially. (g) 1D projections of CX and RE traveling wave dynamics.

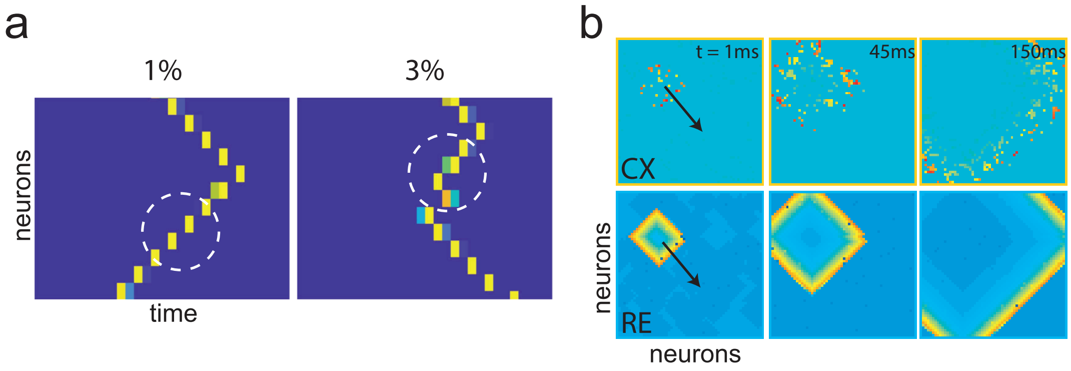

Supplementary Figure S2. (a) 1D projection of thalamic activity, illustrating how lurching behavior of thalamus was lost with different CX→RE connectivity percentages (1% and 3%). A higher percentage of CX→RE connectivity eliminated the time gap between subsequent triggers (highlighted in the white dashed circle) due to cortical inputs. (b) Illustration of the CX and RE traveling waves with overall 25% intracortical connectivity. In this case, the cortical wave was punctate and discontinuous, thereby being difficult to detect.

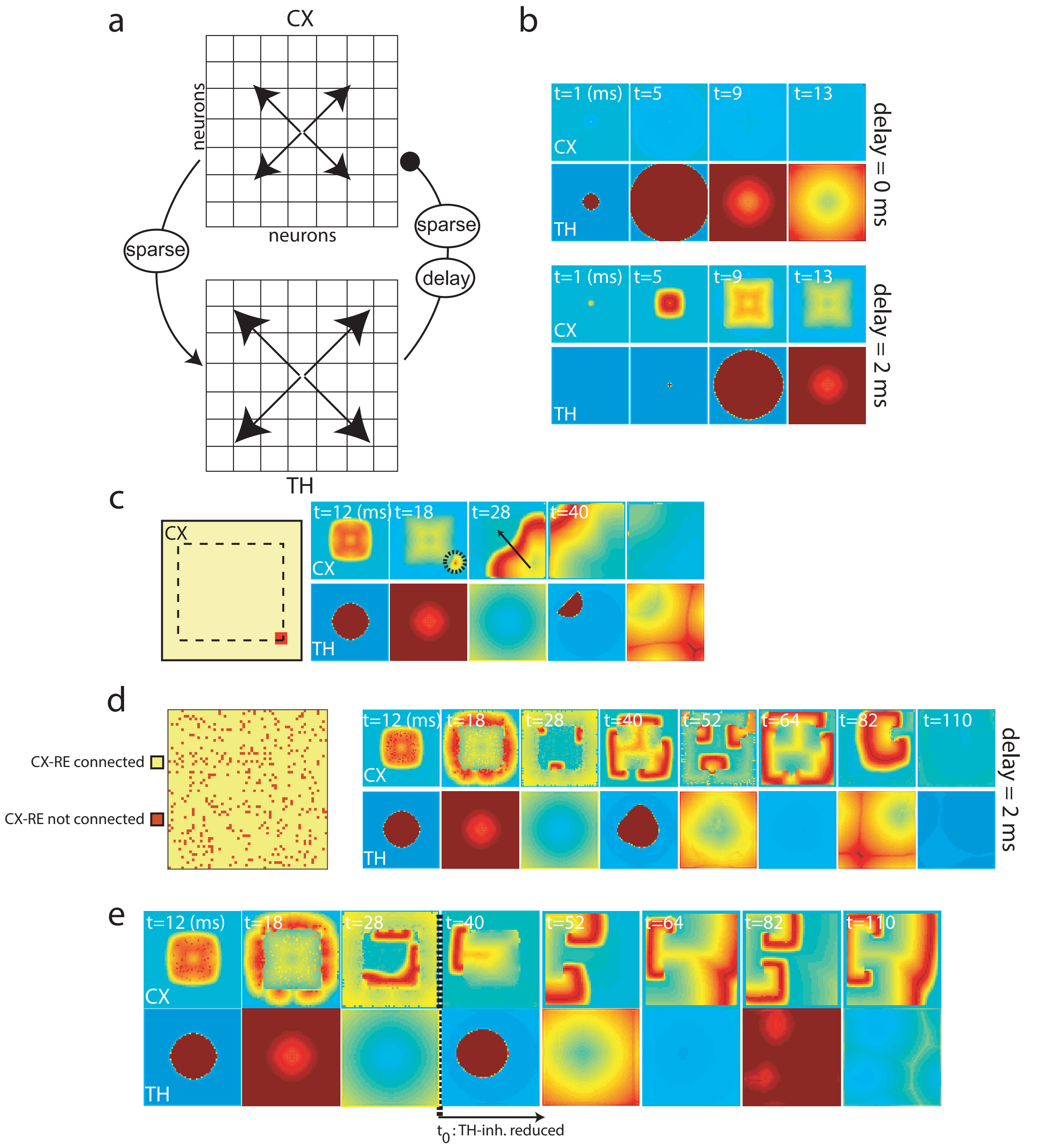

Supplementary Figure S3. (a) The TH-CX model with corticothalamic delay (in comparison with the thalamocortical delay shown in **Fig. 5**a). (b) Effect of different corticothalamic delay parameters on cortical wave patterns. (c) An instance of cortical traveling wave dynamics using a corticothalamic delay of 2 ms. (d) Illustration of diverse cortical and thalamic wave pattern formation using a sparse random connectivity matrix. (e) An instance of generating rotating cortical wave (similar to **Fig. 5**h). TH inhibition was reduced at time t_0.

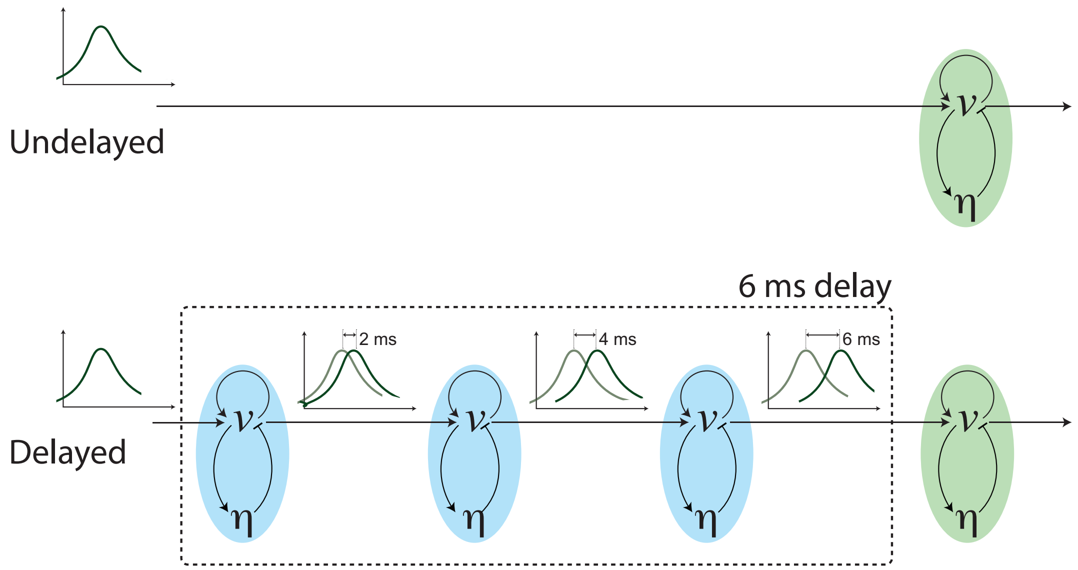

Supplementary Figure S4. Figure illustrating how a synaptic delay was introduced to the system. In a no-delay case (top row), the synaptic input was directly passed to a neuron. To incorporate a delay, additional "fake" neuron modules (blue) were added to delay the synapse before it was fed to the desired neuron (green). Each neuron module added a 2ms delay.

Supplementary Video Legend

Video S1: Closed-loop oscillations of the three-layer thalamocortical system with 60×60 neurons in each layer (99% intracortical connectivity, 99% excitatory neurons, 1% Cortex-Thalamus connections).

Video S2: Video S1 zoomed-in to 10×10 neuron view.

Video S3: Closed-loop spontaneous oscillations of the three-layer thalamocortical system with 60×60 neurons in each layer (99% intracortical connectivity, 80% excitatory neurons, 1% Cortex-Thalamus connections).

Video S4: Video S3 zoomed-in to a 10×10 neuron view.

Video S5: Closed-loop spontaneous oscillations of the three-layer thalamocortical system with 60×60 neurons in each layer (99% intracortical connectivity, 80% excitatory neurons, 3% Cortex-Thalamus connections).

Video S6: Closed-loop oscillations with two highly connected clusters in the cortex (80% excitatory neurons, 10% Cortex-Thalamus connections).

Video S7: Reduced model CX-TH. Dynamics with 90% CX-TH connections and a delay of 2 ms.

Video S8: Reduced model CX-TH. Dynamics with 90% CX-TH connections and a delay of 4 ms.

Video S9: Reduced model CX-TH. Dynamics with one unconnected point of CX-TH (24 ms), which creates a traveling wave in the diagonal direction. Delay of 4 ms.

Video S10: Reduced model CX-TH. Dynamics with a line of unconnected CX-TH points (24 ms), that creates oscillating planar waves. The initial threshold block is to create a unidirectional planar wave ($t < 24$ ms). Delay of 4 ms.

Video S11: Reduced model CX-TH. Dynamics with a line of unconnected CX-TH points (24 ms), that creates oscillating planar waves that are sustained infinitely. Delay is increased from 4 ms to 6 ms at $t = 40$ ms.

Video S12: Reduced model CX-TH. Dynamics with two lines of unconnected CX-TH points (24 ms), which are used to break the planar wave. The wave starts to rotate before being subdued by TH inhibition. Delay of 4 ms.

Video S13: Reduced model CX-TH. Dynamics with two lines of unconnected CX-TH points (24 ms), which are used to break the planar wave. The wave starts to rotate and ultimately evolves into two rotating spirals as the TH inhibition is reduced after $t = 48$ ms. Delay of 4 ms.

Video S14: The same video as Video S11, with an external positive (excitatory) input added at $t = 68$ ms.

Video S15: The same video as Video S11, with an external negative (inhibitory) input added at $t = 68$ ms.

Video S16: Reduced model CX-TH. Dynamics with a line of unconnected CX-TH points (24 ms), that creates traveling waves simulated with a neural array of size 80×80.

Video S17: Closed-loop spontaneous oscillations of the three-layer thalamocortical system with $60{\times}60$ neurons in each layer (99% intracortical connectivity, 90% excitatory neurons, 1% Cortex-Thalamus connections), with RE inhibition increased.

Video S18: Closed-loop spontaneous oscillations of the three-layer thalamocortical system with $60{\times}60$ neurons in each layer (99% intracortical connectivity, 80% excitatory neurons, 1% Cortex-Thalamus connections), with intracortical excitatory weights increased.
